# The IJHPR’s growing scientific impact

**DOI:** 10.1186/s13584-018-0269-1

**Published:** 2018-12-14

**Authors:** Bruce Rosen, Stephen C. Schoenbaum, Avi Israeli

**Affiliations:** 1 Myers-JDC-Brookdale Institute, JDC Hill, Jerusalem, Israel; 2 Josiah Macy Jr. Foundation, New York, NY USA; 3 Hadassah – Hebrew University Medical School, Jerusalem, Israel; 4 Ministry of Health, Jerusalem, Israel

## Abstract

The Israel Journal of Health Policy Research (IJHPR) was launched in 2012, with a mission that included fostering intensive intellectual interactions among health policy scholars in Israel and abroad. Now, as the journal approaches the end of its seventh year of publication, we can all be proud that this component of our mission is increasingly being realized.

As of the end of November 2018, the Web of Science included 404 articles published by the IJHPR. These IJHPR articles had generated 1023 citations via 847 citing articles. Just over 70% of those citing articles were in journals other than the IJHPR, with the vast majority of those being in non-Israeli journals. The authors of the citing articles were most often based in institutions in the US (35%), Israel (33%), England (9%) or Canada (7%).

Looking to the future, we hope that the IJHPR will receive even more submissions from authors based in Israel or other countries that are well-designed data-based studies; thoughtful, comprehensive policy analyses; or important integrations of a body of knowledge. In all instances, these should be relevant to Israeli health policy and health care. We hope that many, ideally most, will also be relevant to scholars, policymakers and professionals in other countries.

The Israel Journal of Health Policy Research (IJHPR) was launched in 2012, with a mission that included fostering intensive intellectual interactions among health policy scholars in Israel and abroad [1]. Now, as the journal approaches the end of its seventh year of publication, we can all be proud that this component of our mission is increasingly being realized.

As of the end of November 2018, the journal had published just over 400 articles. About half of them have been original research articles or integrative articles about Israeli health care, typically by Israeli scholars. The other half have been commentaries by leading scholars, mainly from other countries, which reflect on the international significance of the Israeli studies and/or how the international literature and experience supplements the Israeli studies. As such, the journal explores both what Israel can learn from other countries and what other countries can learn from Israel. As noted in our previous end-of-year editorial [2], the IJHPR is drawing contributions from all of Israel’s university and research centers that are involved in health policy as well as from leading universities in other countries.

In mid-2018 the IJHPR reached an important milestone, with its Journal Citation Report (JCR) impact factor increasing from 1.36 to 1.65. As a result of this increase, the IJHPR’s impact factor is now in the second quartile of all journals in the JCR’s “Public, environmental and occupational health category”.

We are using this year’s end-of-year editorial to share information and insights regarding the IJHPR’s most cited articles. We do so first with regard to the full collection of IJHPR articles published between January 2012 and November 2018. We then provide an analogous presentation that focuses on IJHPR articles published in two recent years: 2016 and 2017.

Of course, citation counts are incomplete measures of a journal’s impact – both in terms of its academic impact and its policy/practice impact. This is especially true of a journal such as the IJHPR which focuses on a particular country and has a policy orientation. In a future editorial we hope to highlight some of the policy impacts of IJHPR articles. With this caveat noted, we are confident that the information below about citation counts and the sources of citations does capture important components of the IJHPR’s impact and that it will be of interest to the IJHPR’s readers.

## Citations and citation leaders for the full IJHPR collection

As of the end of November 2018, the Web of Science included 404 articles published by the IJHPR. These IJHPR articles had generated 1023 citations. These figures are up markedly from those at the end of 2017, at which point the IJHPR had published 339 articles that had generated 770 citations.

The 1023 citations generated by the IJHPR as of November 2018, came from 847 citing articles. Just over 70% of these citing articles were in journals other than the IJHPR, with the vast majority being in non-Israeli journals. The authors of the citing articles were most often based in institutions in the US (35%), Israel (33%), England (9%), or Canada (7%). Other countries accounting for at least 3% each of IJHPR authors’ institutional bases included Australia, Germany, the Netherlands, and China.

Table 1 lists the IJHPR’s all-time most cited articles. Each of them relates to one or more hot topics in health policy: digital health [3], physician-patient communication [3], physician burnout [4], continuity of care [5], quality monitoring [6], technology prioritization [7], tobacco control [8], or medical education [9]. Almost all the seven items on the list were published in 2012-3, with the exception published in 2015 [8]. Three of the seven are data-driven empirical studies [4–6], while the other four are integrative articles [3, 7–9]. All except one [3] are by Israeli authors.

**Table 1:** Most cited IJHPR articles - all years

Citations	Title	Lead author	Publication Year
25	Doctor-patient communication in the e-health era	Jonathan Weiner	2012
21	Compassion fatigue, burnout and compassion satisfaction among family physicians in the Negev area - a cross-sectional study	Nurit El-bar	2013
21	The association between continuity of care in the community and health outcomes: a population-based study	Jacob Dreiher	2012
20	Community healthcare in Israel: quality indicators 2007–2009	Dena Jaffe	2012
17	Which health technologies should be funded? A prioritization framework based explicitly on value for money	Ofra Golan	2012
14	Tobacco policy in Israel: 1948–2014 and beyond	Laura Rosen	2015
14	Medical specialty considerations by medical students early in their clinical experience	Charles Weissman	2012

To give more of a feel of the factors that contribute to citations of articles published by the IJHPR, let us focus for a moment on the IJHPR’s all-time most cited article. Interestingly, unlike the other items on the list it is by a non-Israeli author. The article is “Doctor-Patient Communication in the E-health Era” by Jonathan Weiner of the Johns Hopkins Bloomberg School of Public Health [3]. We have no way of knowing what accounts for the relatively high citation count for this article. Some of the contributing factors could be: the comprehensiveness of the analysis, the focus on not just one, but two, hot topics (the doctor-patient relationship and digital heath), the prominence of its author, and the 2012 publication date. Remarkably, 2018 is the single year in which this article has been cited most often (accounting for 9 of the 25 citations to date).

### Citation and citation leaders for IJHPR articles published in 2016–2017

As of the end of November 2018, the Web of Science included 133 articles published by the IJHPR in 2016–2017. These IJHPR articles had generated 203 citations via 177 citing articles. The authors of the citing articles were most often based in institutions in the US, Israel, England, Canada, or Italy.

Table 2 lists the IJHPR’s most cited articles from among those published in 2016–2017. The topics covered include vaccine hesitancy [10, 11], health care equity [12], medical education [9], the research-policy interface [13], and changing professional boundaries [14] – all topics of current interest in health care policy around the world.

**Table 2:** Most cited articles published in 2016–7

Citations	Title	Lead author
13	Vaccine hesitancy: understanding better to address better	Dewesh Kumar
9	Strengthening the capacities of a national health authority in the effort to mitigate health inequity-the Israeli model	Tuvia Horev
8	Expanding clinical roles for nurses to realign the global health workforce with population needs: a commentary	Claudia Maier
7	Medical education in Israel 2016: five medical schools in a period of transition	Shmuel Reis
7	Views of health system policymakers on the role of research in health policymaking in Israel	Moriah Ellen
7	Vaccine hesitancy as self-determination: an Israeli perspective	Baruch Velan

The most cited IJHPR article from this two-year period is “Vaccine Hesitancy: Understanding Better to Address Better” [10]. It is unusual among IJHPR integrative articles in that it is by a non-Israeli author – Dewesh Kumar. Dr. Kumar is currently working as a Senior Resident in the Department of Community Medicine and Family Medicine at AIIMS (All India Institute of Medical Sciences), Jodhpur, India.

Here, too, although we cannot be sure why this article has generated more citations than other recent IJHPR articles, some of the contributing factors could be: the broad and multi-faceted analytic approach, the appreciation of the complexity of social phenomena, the focus on a topic of major and growing public health concern worldwide, and the serious attention to both to a deeper understanding of a complex social phenomenon and to its policy implications. Interestingly, many of the citing articles explore the relevance of this IJHPR article to particular countries; country names appearing in their titles include Zambia [15], Malaysia [16], Poland [17], Nigeria [18], Australia, Canada, and the UK [19], and (of course) Israel [11].

### Looking to the future

We believe that when the public, persons responsible for health care delivery, and policy-makers have better information about health care and its improvement and about issues underlying existing and potential policies, this will result in better health care and better health policies. We are pleased that the IJHPR has been able to publish a significant number of high-quality articles which have contributed to the global scholarly literature as well as to policy development in Israel and beyond.

Still, we feel that – with your help - the IJHPR could be doing more.

We would like to receive even more submissions from authors based in Israel and other countries that are well-designed data-based studies; thoughtful, comprehensive policy analyses; or important integrations of a body of knowledge. In all instances, these should be relevant to Israeli health policy and health care. We hope that many, ideally most, will also be relevant to scholars, policymakers and professionals in other countries.

## Abbreviation

IJHPR: Israel Journal of Health Policy Research
